# Supplementary material for: MGSurvE: A framework to optimize trap placement for genetic surveillance of mosquito populations
Source: PLoS Comput Biol. 2024 May 6;20(5):e1012046. doi: 10.1371/journal.pcbi.1012046 (PMC11098508; doi:10.1371/journal.pcbi.1012046)
Supplement: S1 Text — A description of the mathematical equations that govern mosquito movement and trap attractiveness implemented as an absorbing Markov chain. (PDF) [file pcbi.1012046.s001.pdf]

MGSurvE: A framework to optimize trap placement for genetic surveillance of mosquito populations
S1 Text: Mathematical Model and Genetic Algorithm Implementation

Héctor M. Sánchez C.[1*], David L. Smith [2,3], John M. Marshall [1]

1 Divisions of Epidemiology and Biostatistics, School of Public Health, University of California, Berkeley, California, United States of America
2 Institute for Health Metrics and Evaluation, University of Washington, Seattle, WA, USA
3 Department of Health Metrics Sciences, School of Medicine, University of Washington, Seattle, WA, USA

* sanchez.hmsc@berkeley.edu

Mathematical Formulation

The default implementation of MGSurvE assumes individuals traverse a landscape following "random-walker" behavior according to a movement matrix (τ), the entries of which, describe the probability of moving from population i to population j in one time step. Incorporating traps as absorbing states within this formulation allows us to calculate outcomes of interest, such as the mean time to absorption (i.e., being trapped) using the properties of absorbing Markov chains.

Movement

The movement weight (α) between two sites ($s_i \to s_j$), is calculated according to an arbitrary shape parameter (κ), which is a function of the distance between them (d), and biological modifying parameters (ρ), which control the shape of the kernel. These parameters are calibrated to expected distances that mosquitoes fly on a daily basis (e.g., the decay rate of an exponential function). To account for resource-directed movement, this shape parameter is then multiplied by the probability of movement (λ), from the source site-type ($\hat{s}_i$) to the destination's site-type ($\hat{s}_j$), which accounts for the expected probabilities that individuals jump from one point-type to another.

$$\alpha(s_i \to s_j) = \kappa(d(s_i \to s_j), \rho) * \lambda(\hat{s}_i, \hat{s}_j) \tag{1}$$

We calculate the movement weights between all the sites in the landscape and normalize the resulting matrix to obtain our migration matrix τ:

$$\tau_{s_n \times s_n} = \begin{pmatrix} \alpha(s_1 \to s_1) & \alpha(s_1 \to s_2) & ... & \alpha(s_1 \to s_{n-1}) & \alpha(s_1 \to s_n) \\ \alpha(s_2 \to s_1) & \alpha(s_2 \to s_2) & ... & \alpha(s_2 \to s_{n-1}) & \alpha(s_2 \to s_n) \\ \vdots & & \ddots & & \vdots \\ \alpha(s_{n-1} \to s_1) & \alpha(s_{n-1} \to s_2) & ... & \alpha(s_{n-1} \to s_{n-1}) & \alpha(s_{n-1} \to s_n) \\ \alpha(s_n \to s_1) & \alpha(s_n \to s_2) & ... & \alpha(s_n \to s_{n-1}) & \alpha(s_n \to s_n) \end{pmatrix} \tag{2}$$

All rows of τ sum to 1, as individuals are limited to move within the s_n set of sites.

Movement with Traps

To calculate the movement matrix incorporating traps as absorbing states (χ), we follow a similar process to calculate the trap probability weights (δ), as we did for the movement weights (α). We determine the movement probability from any given site (s_i) to any given trap (t_j) based on the attractiveness profile ($\hat{\eta}$), and the trap-type attractiveness relative to the current mosquito resource ($\phi(\hat{s_i}, \hat{t_j})$). Equations 4 and 5 are included to mathematically describe that traps are absorbing states:

$$\delta(s_i \to t_j) = \hat{\eta}(d(s_i \to t_j), \hat{\rho}_t) * \phi(\hat{s_i}, \hat{t_j}) \qquad \Rightarrow \nu_{s_n \times t_n} \tag{3}$$

$$\delta(t_i \to s_j || t_i \to t_j | i \neq j) = 0 \qquad \Rightarrow 0_{t_n \times s_n} \tag{4}$$

$$\delta(t_i \to t_i) = 1 \qquad \Rightarrow I_{t_n \times t_n} \tag{5}$$

With these pieces in place, we can define our normalized full movement block matrix with traps as follows, noting that rows must be renormalized to account for possible movement from a site to a trap or to another site:

$$\chi_{(s_n+t_n)\times(s_n+t_n)} = \begin{pmatrix} \tau_{s_n \times s_n} & \nu_{s_n \times t_n} \\ 0_{t_n \times s_n} & I_{t_n \times t_n} \end{pmatrix} \tag{6}$$

Here, τ is our original migration matrix, ν is the probability that a random walker moves from a site to a trap, the null matrix 0 represents the absorbing nature of the traps, and I is the identity matrix stating that walkers who fall into a trap will stay in that trap.

Fitness Function

With the Markov chain transition matrix including absorbing traps in canonical form (χ), we can use the fundamental matrix (F), to calculate a range of Markov chain~~g~~ properties, including the expected number of time steps before an individual is trapped/absorbed. The fundamental matrix is given by:

$$F_{s_n \times s_n} = (I - \tau_{s_n \times s_n})^{-1} \tag{7}$$

The F matrix contains information on how many time steps an individual is expected to spend in site j given that it started in site i, before falling into a trap. Finally, to compute the fitness value (ϕ), we calculate the expected number of time steps (T) before being trapped, when starting in transient site i:

$$T_{s_n \times 1} = F_{s_n \times s_n} * 1_{s_n \times 1} \tag{8}$$

Two metrics that we use in the current paper are: i) the expected number of time steps before being trapped, averaged over all origin sites, i.e., $mean(T_{s_n \times 1})$, and ii) the maximum expected number of time steps before being trapped, considering all origin sites, i.e., $max(T_{s_n \times 1})$. The latter case represents the worst case scenario.

Implementation of the Genetic Algorithm

Genetic Algorithms borrow inspiration from biological inheritance processes to solve optimization problems by generating a "population" of possible solutions (chromosomes) from which the fittest are selected for mating and mutation. By iterating this process over generations, we expect the population to slowly converge upon the optimal region of our problem-space which would correspond to the solution to the task at hand.

Here, we describe how MGSurvE makes use of this approach to solve the optimization problem that can be phrased as: *"Given a heterogeneous environment and a limited number of traps, where should we place the traps?"*.

Terminology When describing our genetic algorithm, the parallels with biological processes might make the description of the methodology confusing. To alleviate this, we provide a list of terms as used in GA literature and specifically in our application:

- Generation: One full discrete cycle of updates (fitness calculation, selection, crossover, mutation) performed upon the current population.
- Population: A group of potential solutions (individuals).
- Individual: Potential solution to the optimization problem with the trap positions being encoded in a genotype.
- Genotype: Way of storing the trap positions (used interchangeably with individual throughout this document).
- Allele: Each slot in our genotype is an allele and contains the information of a trap positions.
- Selection: The process of choosing individuals from the population for mating in order to generate the next generation to be used in the optimization process.
- Mating (crossover): The process of mixing two selected genotypes to produce viable offspring (where viable in this context is an acceptable potential solution to the optimization problem).
- Mutation: Process of altering individual alleles in genotypes in a stochastic way for individuals to be able to explore new potential solutions to the optimization problem.

In the following description of the optimization process, the terms will be used according to these definitions unless stated otherwise.

Optimization Workflow

If we want the algorithm to be able to convert our potential solutions to a fitness (or cost) that can be optimized, we need to define our problem in a form that is compatible with genetic algorithm approaches. In our case this is relatively simple, as we encode the positions of the traps into the GA's chromosomes as depicted in figure 1. For the continuous case, we store the trap positions directly into pairs of alleles, whereas in the discrete case, we store the ID of the site in which the traps would be positioned.

With this problem mapping at hand, we can go on to describe the set of instructions to run at each generation (optimization step) of our genetic algorithm:

1. Fitness Calculation: Each individual in the population of potential solutions is evaluated according to the calculations described in the "Mathematical Formulation" section of this document. Specifically, we update the values for equations 3 to 8.

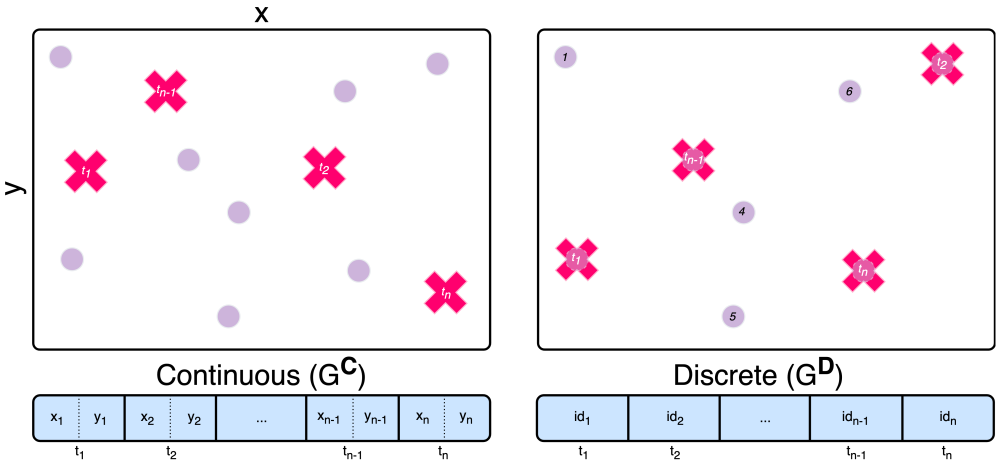

Fig 1. Chromosome-to-trap-position mapping. Continuous optimization chromosomes store trap (magenta crosses) coordinates in pairs of alleles (x/y or lon/lat), while discrete optimization chromosomes store the id of the site (purple circles) at which they are located.

2. Individual Selection: A sub-sample of the population is selected for mating (fitter individuals have higher probabilities to get selected).
3. Crossover (Mating): Individuals in the new pool are paired for their chromosomes to be combined for the next generation's offspring.
4. Mutation: The generated offspring is subject to random variations in their chromosomes in hopes that some of these changes will lead to better solutions.

In this section, we will go through the details of each step and how the base implementation of MGSurvE goes through each step (for both, discrete and continuous optimization tasks) in an effort to find better solutions to trapping random walkers as quickly as possible by iterating over the potential positions of the traps.

Fitness Calculation

The fitness (or cost) function is the quantity that guides the artificial evolutionary process, and is the factor we will try to maximize or minimize. MGSurvE computes a summary statistic on the time it would take for a random walker in our network to fall into an absorbing site (a trap), given that it started from any site in our landscape (Equation 8). To compute these statistics on our chromosomes, we follow this procedure:

1. If the chromosome is discrete, place the traps in the positions of the matching IDs of the sites stored in each allele of the potential solution.
2. With each of the traps' attraction kernels (δ), their positions, and the base migration matrix (τ), we update our full movement block matrix (Equation 6).
3. With this updated full movement block matrix and renormalized migration τ, we calculate the expected number of time-steps before being trapped starting from each site in the landscape (Equation 8).
4. Finally, to calculate our fitness, we apply a summary statistic function to these set of expected time-steps (mean for a balanced placement over the landscape, max to prioritize the most remote parts of the geography).

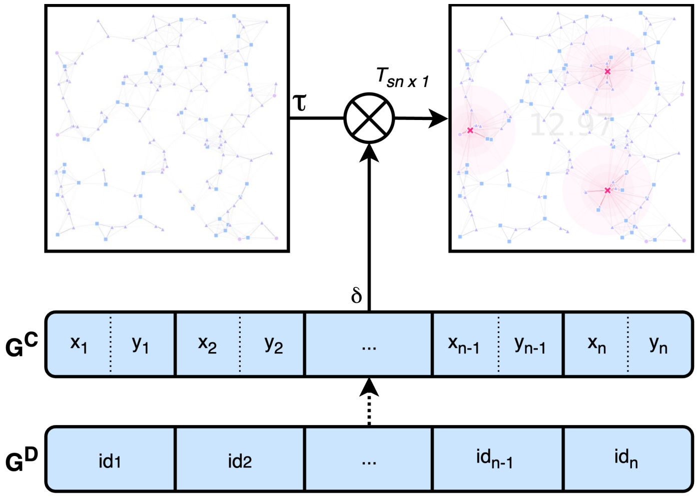

Fig 2. Chromosome to fitness calculation. If our chromosome is discrete (G^D) we place the traps in the position of the sites which correspond to the IDs stored in it so that we have a representation that is equivalent to the continuous one (G^C). To get the fitness, we calculate Markov's Fundamental Matrix (eq. 8) with the migration matrix (τ), and the traps positions with their attraction kernels (δ); so that we obtain a summary statistic of the time it would take for a random walker to fall into one of the absorbing states.

This process is summarized in Fig 2, and repeated for each chromosome in our current GA population. Once we have the fitness for each individual in the population, we can go on to the selection step of the algorithm.

Selection

The selection process involves choosing individuals in a scheme that favors the fittest entities from our pool in order to allow them to populate the next generation of solutions in our evolutionary process. MGSurvE is compatible with any of the DEAP selection operators but uses the tournament operator by default. In its stock implementation, this process randomly takes a defined number of tournament participants (tournament size) from the initial population, selects the fittest from them and repeats the process until we have the desired population size in our next generation (Fig 3).

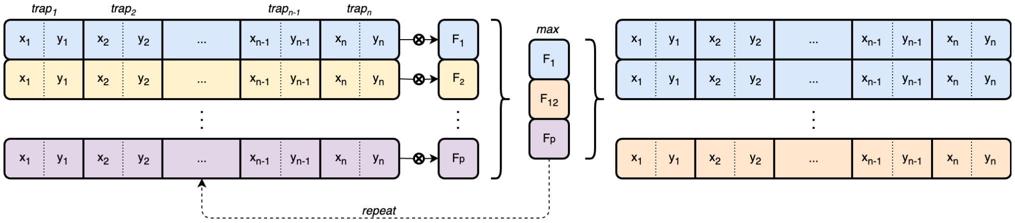

Fig 3. Selection. Genotypes are selected randomly from the initial population (left) based and compared in their fitness scores (middle). The one with the best score in the selected grouped is copied to the new population (right). The process is repeated until enough individuals have been selected to populate the next generation of our algorithm.

Crossover

This step involves taking pairs of chromosomes and "mixing" their alleles to simulate biological mating processes. The driving idea behind this process is that parent solutions with good fitness values could produce better-fitted offspring through combination of their genotypes. MGSurvE provides extensions to DEAP's `cxBlend` crossover operation for continuous optimization, in which a random subset of the paired alleles are averaged out between the two parents; along with `cxUniform` for discrete optimization tasks, in which alleles are swapped between the two parents to generate the offspring (Fig 4).

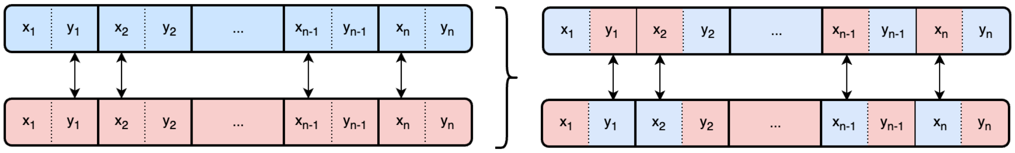

Fig 4. Crossover. Pairs of genotypes are randomly selected, and their alleles are mixed together (arrows) in order to generate offspring in hopes that they have better solutions to the optimization task.

Mutation

The final step in our evolutionary process is to allow the alleles of selected individuals in our population to "mutate" parts of their chromosomes to allow for new possible solutions to be generated. This allows the evolutionary process to explore the vicinity of the original chromosome in the solution space by modifying values of their alleles (Fig 5). Our base MGSurvE implementation provides extensions to the `mutGaussian` operator for continuous optimization operations (where an allele is modified with a normal distribution centered at the allele's value) along with a random uniform allele replacement one for discrete tasks (where alleles are swapped with other possible sites IDs in the landscape).

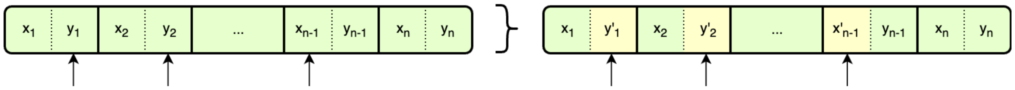

Fig 5. Mutation. Individuals are selected randomly for mutation, process in which some of their alleles' values are modified (arrows) so that the algorithm can explore for novel solutions.

Solution Selection and Evaluation

As with any stochastic optimization task, we need to run our optimization routines several times to be able to evaluate the performance of our algorithm. This helps us to both notice if it is getting stuck in local optima, and to explore potentially better solutions (as the starting points in solution space will be different in each iteration). One of the ways to check how the algorithm is performing is to plot the evolution of the best solution of each one of the iterations across the GAs generations (Fig 6). Some of the problems that can be detected with these plots include:

- Lack of exploration: If our plot is constantly flat-lining, we might need to increase our mutation and crossover rates to allow for more exploration. Another

option is to lower the tournament size in the selection algorithm or increasing the population size.

- Solution instability: If our plot is jumping up and down, our mutation and crossover rates are probably too high, so we are losing our best solutions often.
- Running the algorithm for few generations: When our algorithm is not run for enough generations, our plot will not have been stabilized to any value in the plot. We can correct this by simply running the algorithm for longer generation spans.
- Widely-varying starting points: If our first generations start at very different points in terms of fitness, we might need to increase our population size.

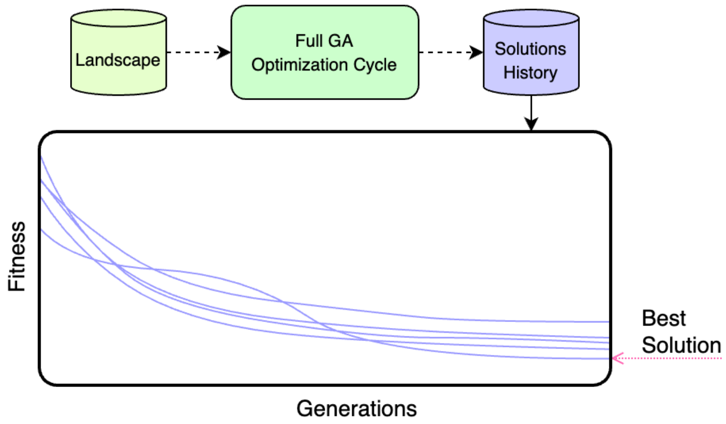

Fig 6. Solution selection and evaluation. By plotting the evolution of the system we can check and diagnose potential problems with our parameters and algorithm.

Parameter Selection

In terms of avoiding local optima, that is a complex topic which, unfortunately, comes down to a combination of rules of thumb and trial and error. In our specific domain, and after lots of benchmarks and tests, we settled for the following initial "auto" parameters for the GA optimization routines. Here, we explain their use, default values, and provide some information on what to expect if they are modified (parameters marked with asterisk "*" only apply to the continuous optimization case):

- *population size (popSize=12.5 times the initial number of traps)*: This value controls the number of potential solutions (genotypes) at each GA generation. If this parameter is too big, many solutions can be evaluated at once, but a lot of computer memory has to be allocated to storing the genotypes; in contrast, if it is too small the algorithm will be slow or incapable of finding optimum solutions as exploration would be limited.
- *genotype mating probability (mate=0.3)*: Probability of any potential solution to be selected for mating. If this value is too high, the algorithm might not converge but if it's too slow it might take a long time to generate good results.
- *allele mating crossover rate (cxpb=0.5)*: Probability of each allele in a pair of solutions selected for crossover. If this value is too high, the algorithm might not converge.

- *allele mating blend* (alpha=0.5)*: In continuous optimization, when this value is set to 1/2, the alleles selected from the genotypes are averaged out to produce offspring. No explorations on this parameter were made and should probably not be changed from the default.
- *genotype mutation probability (mutpb=0.4)*: Probability of each genotype to be selected for mutation process. Lowering this value too much leads to the algorithm not being able to explore new solutions, while increasing it too much hinders the stability of the GA.
- *allele mutation probability (ipb=0.5)*: Individual allele's probability to be selected for mutation. Higher values favor exploration at the expense of solution stability.
- *allele mutation center* (mean=0)*: For continuous optimization, using a value of zero centers a random normal distribution around the current value of the allele. No explorations on this parameter were made and it should be kept at default to avoid solutions to "creep out" of the landscape's bounding box.
- *allele mutation deviation* (sd=1/2.5 times the difference between the maximum span in the landscape's bounding box)*: For continuous optimization, it determines the standard deviation on the normal distribution from which the mutation value is drawn. Higher values lead to more exploration but lower solution stability, while lower values make it more difficult to explore for new solutions.
- *population tournament size (tSize=3)*: This integer controls the number of genotypes grabbed for comparison and selection of the one with the highest fitness to populate the next generation of solutions. If this value is set too high the algorithm could get stock in local optima easily.
