# Supplementary material for: MGSurvE: A framework to optimize trap placement for genetic surveillance of mosquito populations
Source: PLoS Comput Biol. 2024 May 6;20(5):e1012046. doi: 10.1371/journal.pcbi.1012046 (PMC11098508; doi:10.1371/journal.pcbi.1012046)

MGSurvE: A framework to optimize trap placement for genetic surveillance of mosquito populations
S2 Text: Urban and Semi-Rural Landscape Code

Héctor M. Sánchez C.[1*], David L. Smith [2,3], John M. Marshall [1]

1 Divisions of Epidemiology and Biostatistics, School of Public Health, University of California, Berkeley, California, United States of America
2 Institute for Health Metrics and Evaluation, University of Washington, Seattle, WA, USA
3 Department of Health Metrics Sciences, School of Medicine, University of Washington, Seattle, WA, USA

* sanchez.hmsc@berkeley.edu

In this document, we provide the PDF version of the jupyter notebooks available in our github repository (https://github.com/Chipdelmal/MGSurvE) for users to be able to replicate the analyses presented in this document. These demonstrations are available from our Zenodo release 10.5281/zenodo.8342531 onwards, as well as in our github repository at the following URLs:

- São Tomé: https://github.com/Chipdelmal/MGSurvE/blob/main/MGSurvE/demos/STP/STP-Discrete.ipynb
- Yorkeys Knob: https://github.com/Chipdelmal/MGSurvE/blob/main/MGSurvE/demos/YKN/YKN.ipynb

Additionally, these interactive notebooks are available in a docker container as part of our 2023 Live Webinar (https://github.com/Chipdelmal/MGSurvE_Webinar2023), which contains materials and live explanations of the software available on YouTube (https://youtu.be/RhYmeJ3XZ_8?si=vmy1MmYO6XRFGxlb).

São Tomé

This demonstration follows closely one of the ones shown in our preprint. In this application we will set to two fixed traps and import an externally-generated migration matrix. We will begin by loading all the required libraries and packages:

```
# Fix PROJ path
---------------------------------------------------------------
import os;
os.environ['PROJ_LIB']=r'/opt/conda/pkgs/proj-9.2.1-ha5fc9e9_0/share/
proj'
# Load libraries
--------------------------------------------------------------
from os import path
import numpy as np
import pandas as pd
import cartopy.crs as ccrs
import matplotlib.pyplot as plt
from sklearn.preprocessing import normalize
import MGSurvE as srv
import warnings
warnings.filterwarnings("ignore")
```

Landscape

We will now setup some of the experiment's constants. In this case we will setup the experiment to 7 traps (`TRPS`) by default with the option to set two traps as fixed (`FXD_TRPS`) as `True`. These fixed traps will be located in two small landmasses at the north and south of the main São Tomé landscape. The experiment id `ID` is just a label for the output files, with the run id `RID` being used for iterations of the stochastic optimization process; and the `IX_SPLIT` used to separate to separate sites in São Tomé from the ones in the island of Príncipe. Finally, we will create a folder to export our results into.

```
(TRPS_NUM, FXD_TRPS) = (12, True)
# Experiment ID (RID) and stochastic iteration id (RID)
-----------------------
(ID, RID) = ('STP', 0)
IX_SPLIT = 27
# Output folder
---------------------------------------------------------------
OUT_PTH = './out/'
srv.makeFolder(OUT_PTH)
```

We need to read our points coordinates. These positions are stored in a CSV file as *longitude/latitude* format, so we will load them into a pandas dataframe and set them to the same point-type (`t`):

```
sites = pd.read_csv(path.join('../../data/', 'STP_LatLonN.csv'))
sites['t'] = [0]*sites.shape[0]
```

Our original CSV file contains the information for both São Tomé and Príncipe, but in this example we will be focusing on the main São Tomé land, so we will split the dataframe to get the positions we are interested on (`SAO_TOME_LL`). At the same time, we will get the landscape bounding box (`SAO_bbox`), center (`SAO_cntr`), and the ID of the nodes where we want our fixed traps to be in (`SAO_FIXED`):

```
# Get longitude-latitudes for main island
-------------------------------------
SAO_TOME_LL = sites.iloc[IX_SPLIT:]
# Get bounding box
------------------------------------------------------------
SAO_bbox = (
    (min(SAO_TOME_LL['lon']), max(SAO_TOME_LL['lon'])),
    (min(SAO_TOME_LL['lat']), max(SAO_TOME_LL['lat']))
)
# Get centroid
----------------------------------------------------------------
SAO_cntr = [i[0]+(i[1]-i[0])/2 for i in SAO_bbox]
SAO_LIMITS = ((6.41, 6.79), (-0.0475, .45))
# Get sites IDs for fixed traps
-----------------------------------------------
SAO_FIXED = [51-IX_SPLIT, 239-IX_SPLIT]
FXD_NUM = len(SAO_FIXED)
```

We now import the migration matrix and split it to get the São Tomé subset of transitions (`SAO_TOME_MIG`):

```
migration = np.genfromtxt(
    path.join('../../data/', 'STP_MigrationN.csv'), delimiter=','
)
# Spliting up Sao Tome and making sure rows are normalized
--------------------
msplit = migration[IX_SPLIT:,IX_SPLIT:]
SAO_TOME_MIG = normalize(msplit, axis=1, norm='l1')
```

We now setup the traps. In this case we will set all the traps to the same type `0`. Generally speaking, the initial position in terms of longitud and latitude is not important in this case as we will be doing discrete optimization, but the current version of MGSurvE still needs some positions vector to be provided (will likely get deprecated in future releases). Something that is important, however, is that we setup our fixed traps in the right node id for them to be located in the small masses of land at the north and south of the island (`SAO_FIXED`), so we setup a vector of a number of traps that can be placed at whichever node we want (`0`, in this case), and two traps that are located in specific nodes (`SAO_FIXED` which are `[24, 212]`).

```
(initTyp, initFxd) = ([0]*TRPS_NUM, [0]*TRPS_NUM)
if FXD_TRPS:
    initFxd = ([0]*(TRPS_NUM-FXD_NUM) + [1]*FXD_NUM)
# Initialize the position of traps (not strictly needed)
-----------------------
(initLon, initLat) = ([[SAO_cntr[0]]*TRPS_NUM,
[SAO_cntr[1]]*TRPS_NUM])
# Initialize traps to nodes IDs
-------------------------------------------------
sid = [0]*(TRPS_NUM-FXD_NUM) + SAO_FIXED
```

With this in place, we can setup our trap kernel and our traps dataframe:

```
tKer = {0: {'kernel': srv.exponentialDecay, 'params': {'A': 0.5, 'b':
0.041674}}}
# Initialize traps
--------------------------------------------------------------
traps = pd.DataFrame({
    'sid': sid, 'lon': initLon, 'lat': initLat,
    't': initTyp, 'f': initFxd
})
```

We can now initialize our landscape with the info we gathered from our previous steps:

```
lnd = srv.Landscape(
    SAO_TOME_LL, migrationMatrix=SAO_TOME_MIG,
    traps=traps, trapsKernels=tKer,
    landLimits=SAO_LIMITS
)
bbox = lnd.getBoundingBox()
trpMsk = srv.genFixedTrapsMask(lnd.trapsFixed)
```

And let's plot our traps and landscape now!

```
# Plot Traps Kernels
------------------------------------------------------------
(fig, ax) = plt.subplots(1, 1, figsize=(15, 5), sharey=False)
(fig, ax) = srv.plotTrapsKernels(fig, ax, lnd, distRange=(0, 100),
aspect=.175)
ax.set_title("Traps Kernel")
ax.set_xlabel("Distance (m)")
ax.set_ylabel("Trapping Probability")
fig.savefig(
    path.join(OUT_PTH, '{}D-{:02d}-{:02d}_KER.png'.format(ID,
TRPS_NUM, RID)),
    facecolor='w', bbox_inches='tight', pad_inches=0.1, dpi=300
)
# Plot Landscape
```

```
--------------------------------------------------------------
(fig, ax) = (
    plt.figure(figsize=(15, 15)),
    plt.axes(projection=ccrs.PlateCarree())
)
lnd.plotSites(fig, ax, size=250)
lnd.plotLandBoundary(fig, ax)
srv.plotClean(fig, ax, bbox=lnd.landLimits)
fig.savefig(
    path.join(OUT_PTH, '{}_{:02d}_CLN.png'.format(ID, TRPS_NUM)),
    facecolor='w', bbox_inches='tight', pad_inches=0.1, dpi=300
)
```

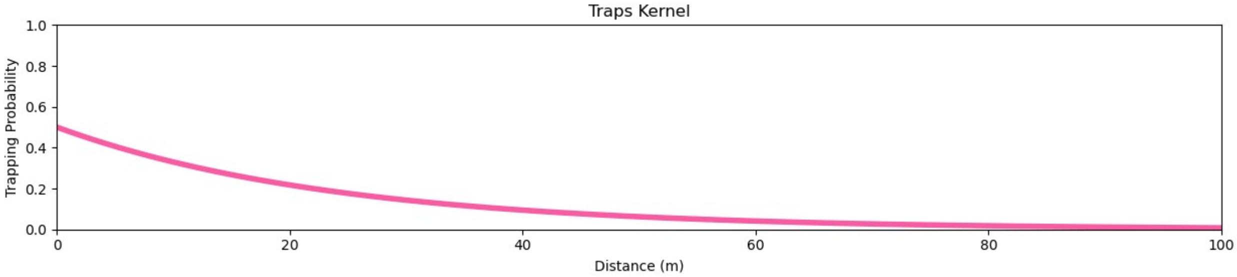

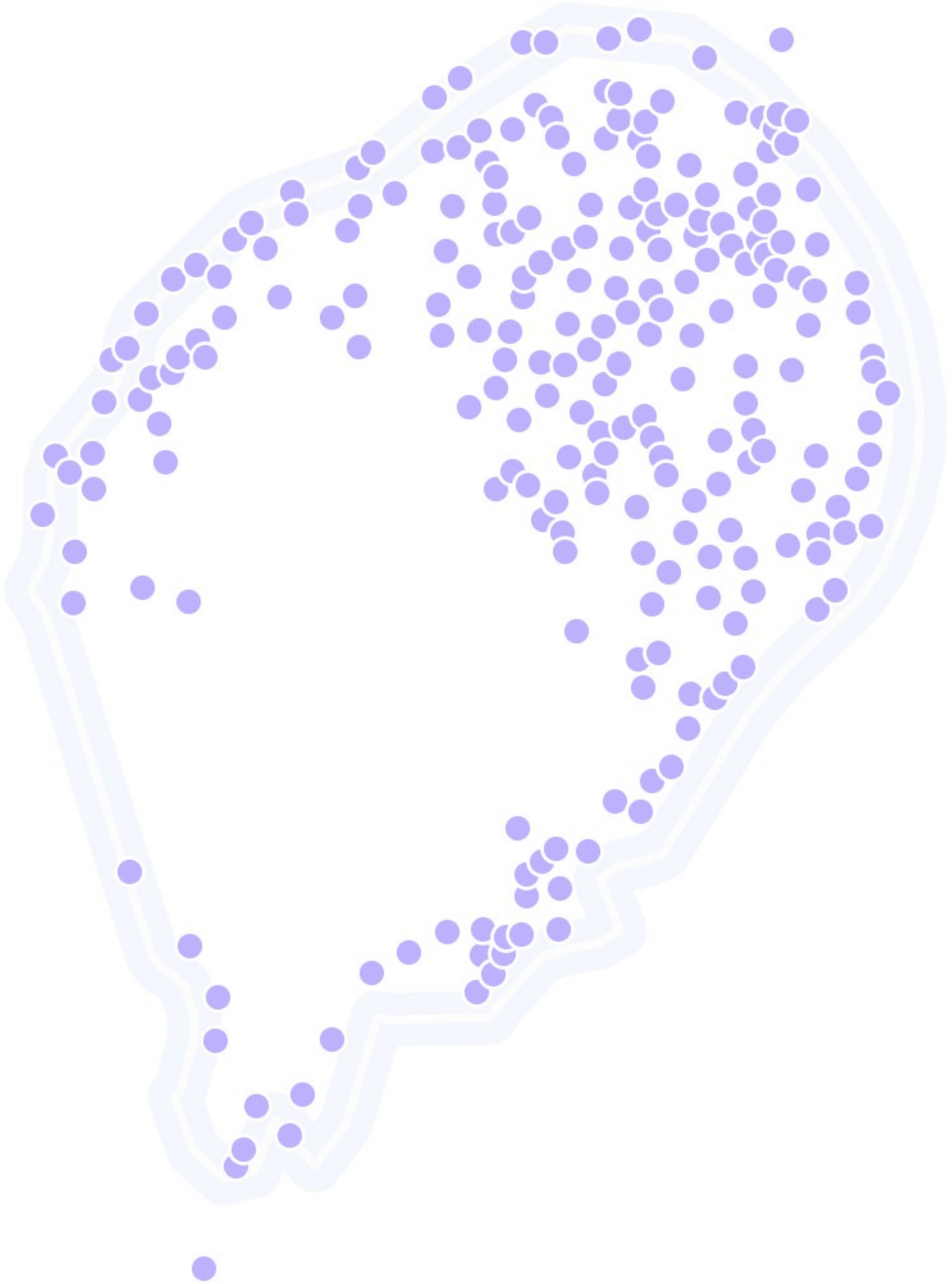

Optimization

We now turn to the optimization section of our code. For this demo, let's setup the summary statistic for our fitness function as the mean of the expected times for mosquitoes to fall into traps (`np.mean`). We will use some mutation, crossover, and selection parameters that we have found useful by trial and error. Some general thoughts on the selection of these parameters, though:

- We want the population size to grow proportionally with the number of variables to optimize, but keeping it as low as possible so that it's not very computationally intensive, and allows for exploration.
- The mutation probability of each potential solution (`mutpb`) should be high enough to allow for exploration but not so large that the optimization fails to converge (same goes for the probability of mutation of each individual allele in a chromosome `indpb`).
- The crossover probability between potential solutions (`cxpb`) allows for the exchange of alleles between good solutions in hopes that the mix results in a better fitness value, so we should have this value as high as we want but not so much that we are sampling too often.

```
(GENS, GA_SUMSTAT, VERBOSE) = (1000, np.mean, False)
# Parameters for the genetic algorithm internal operation
---------------------
POP_SIZE = int(10*(lnd.trapsNumber*1.5))
(MAT, MUT, SEL) = (
    {'cxpb':  0.300, 'indpb': 0.35},
    {'mutpb': 0.375, 'indpb': 0.50},
    {'tSize': 3}
)
```

With that, we can now instantiate and run our GA algorithm! Keep in mind that it will take some time to finish, but look at the `min` column, which should be slowly getting smaller with each iteration (generation) of the algorithm:

```
outer = GA_SUMSTAT
(lnd, logbook) = srv.optimizeDiscreteTrapsGA(
    lnd, pop_size=POP_SIZE, generations=GENS, verbose=VERBOSE,
    mating_params=MAT, mutation_params=MUT, selection_params=SEL,
    fitFuns={'inner': np.sum, 'outer': outer}
)
```

And let's export our results to disk for future use:

```
srv.exportLog(logbook, OUT_PTH, '{}D-{:02d}-{:02d}_LOG'.format(ID,
TRPS_NUM, RID))
srv.dumpLandscape(lnd, OUT_PTH, '{}D-{:02d}-{:02d}_TRP'.format(ID,
TRPS_NUM, RID), fExt='pkl')
```

Analysis

For this demonstration, we will plot the evolution of our genetic algorithm in finding the most optimum solution. The region shaded in blue spans the best solution (bottom) to the worst one (top), with the mean being plotted in white somewhere between these values. For a GA it's important that the best solution doesn't bounce around too much and that it keeps improving as generations go on, but it's also important to have some variability so that exploration is taking place, so a reasonably wide envelope is generally a good sign for our applications:

```
log = pd.DataFrame(logbook)
log.rename(columns={'median': 'avg'}, inplace=True)
(fig, ax) = plt.subplots(1, 1, figsize=(15, 5), sharey=False)
ax.set_title("GA Optimization")
ax.set_xlabel("Fitness")
ax.set_ylabel("Generations")
srv.plotGAEvolution(
    fig, ax, log,
    colors={'mean': '#ffffff', 'envelope': '#1565c0'},
    alphas={'mean': .75, 'envelope': 0.5},
    aspect=1/3
)
fig.savefig(
    path.join(OUT_PTH, '{}D-{:02d}-{:02d}_GA.png'.format(ID, TRPS_NUM,
RID)),
    facecolor='w', bbox_inches='tight', pad_inches=0.1, dpi=300
)
```

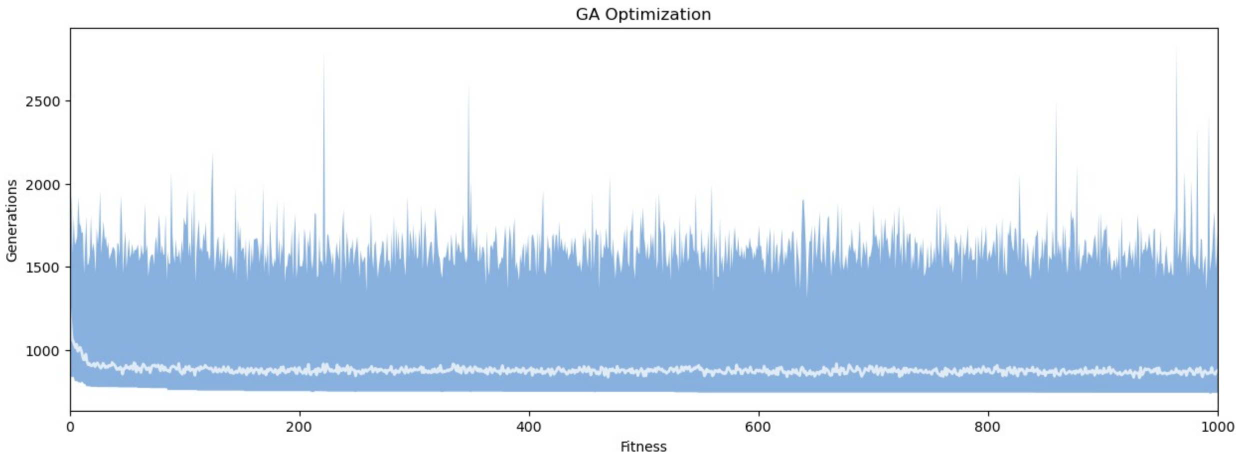

Finally, let's plot our optimized landscape!

```
lnd = srv.loadLandscape(
    OUT_PTH, '{}D-{:02d}-{:02d}_TRP'.format(ID, TRPS_NUM, RID),
    fExt='pkl'
)
(fig, ax) = (
```

```
    plt.figure(figsize=(15, 15)),
    plt.axes(projection=ccrs.PlateCarree())
)
lnd.plotSites(fig, ax, size=250)
lnd.plotTraps(
    fig, ax,
    zorders=(30, 25), transparencyHex='55',
    proj=ccrs.PlateCarree()
)
srv.plotClean(fig, ax, bbox=lnd.landLimits)
lnd.plotLandBoundary(fig, ax)
srv.plotClean(fig, ax, bbox=lnd.landLimits)
fig.savefig(
    path.join(OUT_PTH, '{}D-{:02d}-{:02d}_TRP.png'.format(ID,
TRPS_NUM, RID)),
    facecolor='w', bbox_inches='tight', pad_inches=0.1, dpi=400
)
```

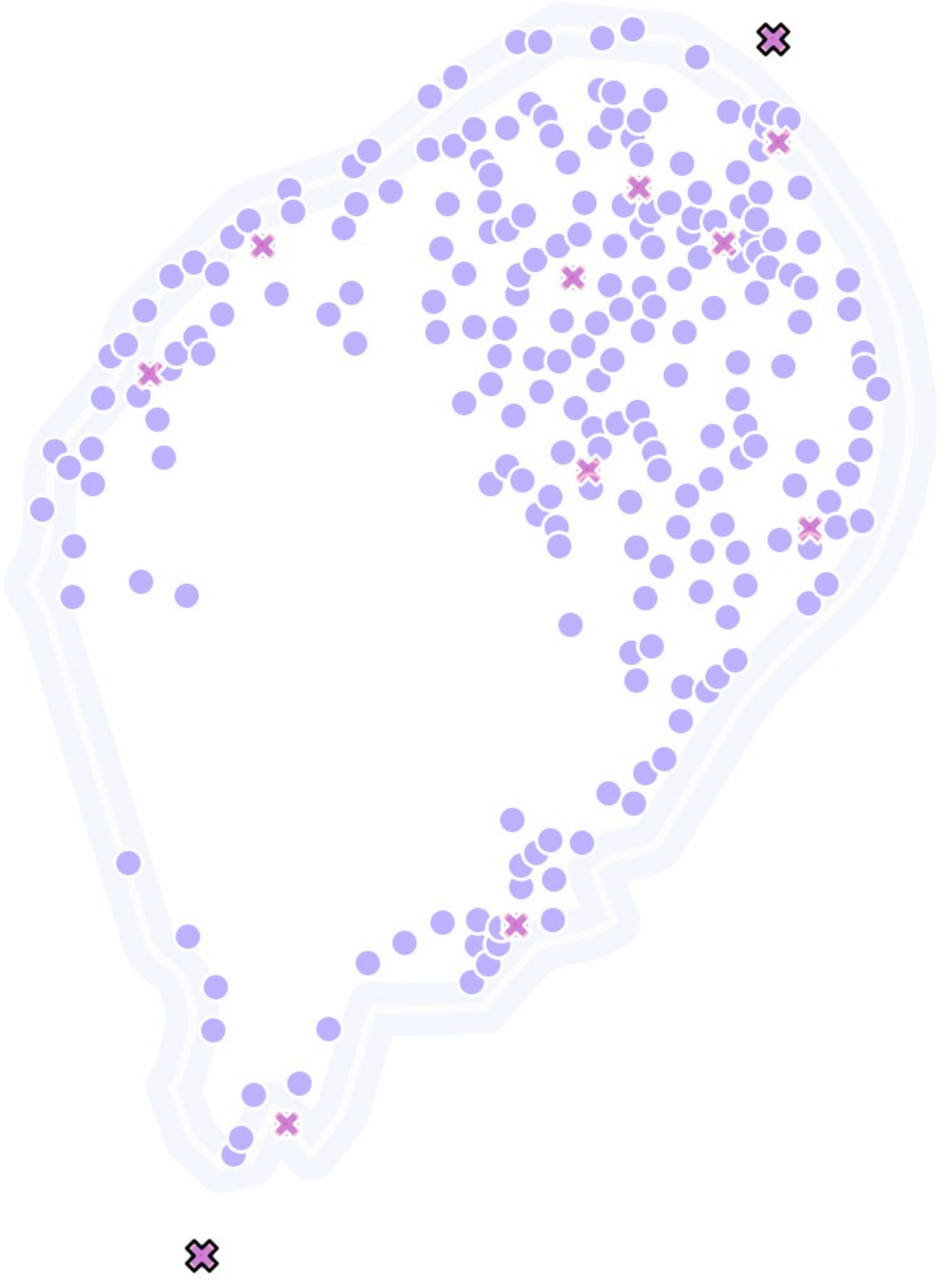

Yorkeys Knob

This demonstration follows closely one of the ones shown in our preprint. We will begin by loading all the required libraries and packages:

```
from os import path
import numpy as np
import pandas as pd
import cartopy.crs as ccrs
import matplotlib.pyplot as plt
import MGSurvE as srv
import warnings
warnings.filterwarnings("ignore")
```

Landscape

Let's get some constants set for our experiments. The number of traps will be controlled by `TRPS_NUM`, whith experiment id `ID` being just a label for the output files, and the run id `RID` being used for iterations of the stochastic optimization process. All of our files will be exported to the `sims_out` folder on the root of the current directory.

```
TRPS_NUM = 16
# Experiment ID (RID) and stochastic iteration id (RID)
-----------------------
(ID, RID) = ('YKN', 0)
# Output folder
---------------------------------------------------------------
OUT_PTH = './out/'
srv.makeFolder(OUT_PTH)
```

To get started with our landscape, we need to import our latitude/longitude pairs for the households in it. Once we have loaded them into a pandas dataframe (`YK_LL`), we set the type `t` for all of the traps as `0`, as we will be assuming that each household contains all the required resources for mosquito survival. Finally, we will define a bounding box (`YK_BBOX`) for plotting purposes:

```
LND_PTH = '../../data/{}_LatLon.csv'.format(ID)
YK_LL = pd.read_csv(LND_PTH, names=['lon', 'lat'])
# Set all points to the same type
---------------------------------------------
YK_LL['t'] = [0]*YK_LL.shape[0]
# Create a bounding box for data plotting
-------------------------------------
pad = 0.00125
YK_BBOX = (
    (min(YK_LL['lon'])-pad, max(YK_LL['lon'])+pad),
```

```
    (min(YK_LL['lat'])-pad, max(YK_LL['lat'])+pad)
)
```

And we will load our *Aedes aegypti* mosquito movement kernel:

```
mKer = {
    'kernelFunction': srv.zeroInflatedExponentialKernel,
    'kernelParams': {'params': srv.AEDES_EXP_PARAMS, 'zeroInflation':
1-0.28}
}
```

This time we will be doing two trap types (`TRAP_TYP`). Half of them will be assigned to type `0` with an exponential decay profile; and the remaining ones will be assigned to type `1`, with a sigmoid attractiveness kernel. With this, we can initialize our traps dataframe `traps`:

```
TRAP_TYP = [0]*int(TRPS_NUM/2) + [1]*(TRPS_NUM-int(TRPS_NUM/2))
# Dummy initialization positions
----------------------------------------------
cntr = ([np.mean(YK_LL['lon'])]*TRPS_NUM,
[np.mean(YK_LL['lat'])]*TRPS_NUM)
# Traps initialization DataFrame
----------------------------------------------
traps = pd.DataFrame({
    'sid': [0]*TRPS_NUM, 'lon': cntr[0], 'lat': cntr[1],
    't': TRAP_TYP, 'f': [0]*TRPS_NUM
})
```

And we now define the shapes of our traps attractiveness kernels accordingly:

```
tKer = {
    0: {
        'kernel': srv.exponentialDecay,
        'params': {'A': 0.5, 'b': 0.0629534}
    },
    1: {
        'kernel': srv.sigmoidDecay,
        'params': {'A': 0.5, 'rate': .25, 'x0': 1/0.0629534}
    }
}
```

With all this in place, we can initialize our landscape object:

```
lnd = srv.Landscape(
    YK_LL,
    kernelFunction=mKer['kernelFunction'],
kernelParams=mKer['kernelParams'],
    traps=traps, trapsKernels=tKer, trapsRadii=[0.250, 0.125, 0.100],
    landLimits=YK_BBOX
```

```
)
bbox = lnd.getBoundingBox()
```

Let's now plot a sample of the relative mosquito movement kernel (the red vertical line at distance 0 represents the zero-inflation of the movement kernel), the traps attractiveness, and our landscape!

```
# Mosquito movement kernel
-----------------------------------------------------
(maxRange, samples) = (100, 10)
x = np.array(list(np.arange(0.15, maxRange, maxRange/samples)))
(fig, ax) = plt.subplots(1, 1, figsize=(15, 5), sharey=False)
(fig, ax) = srv.plotMovementKernel(fig, ax, x, lnd)
ax.set_ylim(0, 1)
ax.set_title(f'Mosquito relative movement kernel ({samples} samples)')
# Traps kernels
----------------------------------------------------------------
(fig, ax) = plt.subplots(1, 1, figsize=(15, 15), sharey=False)
(fig, ax) = srv.plotTrapsKernels(
    fig, ax, lnd, distRange=(0, 50), aspect=.25
)
ax.set_title('Traps kernels')
# Landscape
--------------------------------------------------------------------
(fig, ax) = (
    plt.figure(figsize=(15, 15)),
    plt.axes(projection=ccrs.PlateCarree())
)
lnd.plotSites(fig, ax, size=50)
srv.plotClean(fig, ax, bbox=lnd.landLimits)
```

```
(<Figure size 1500x1500 with 1 Axes>, <GeoAxesSubplot:>)
```

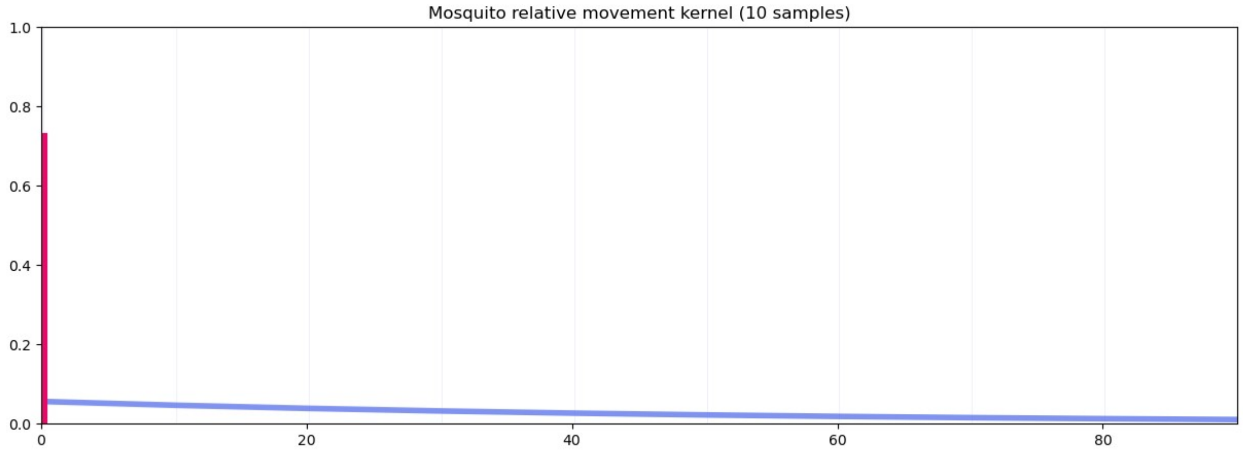

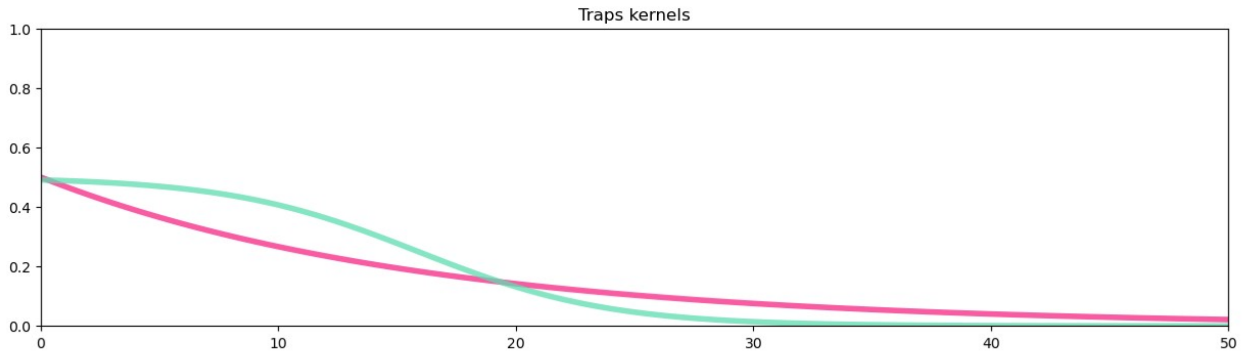
Traps kernels
1.0
0.8
0.6
0.4
0.2
0.0
0
10
20
30
40
50

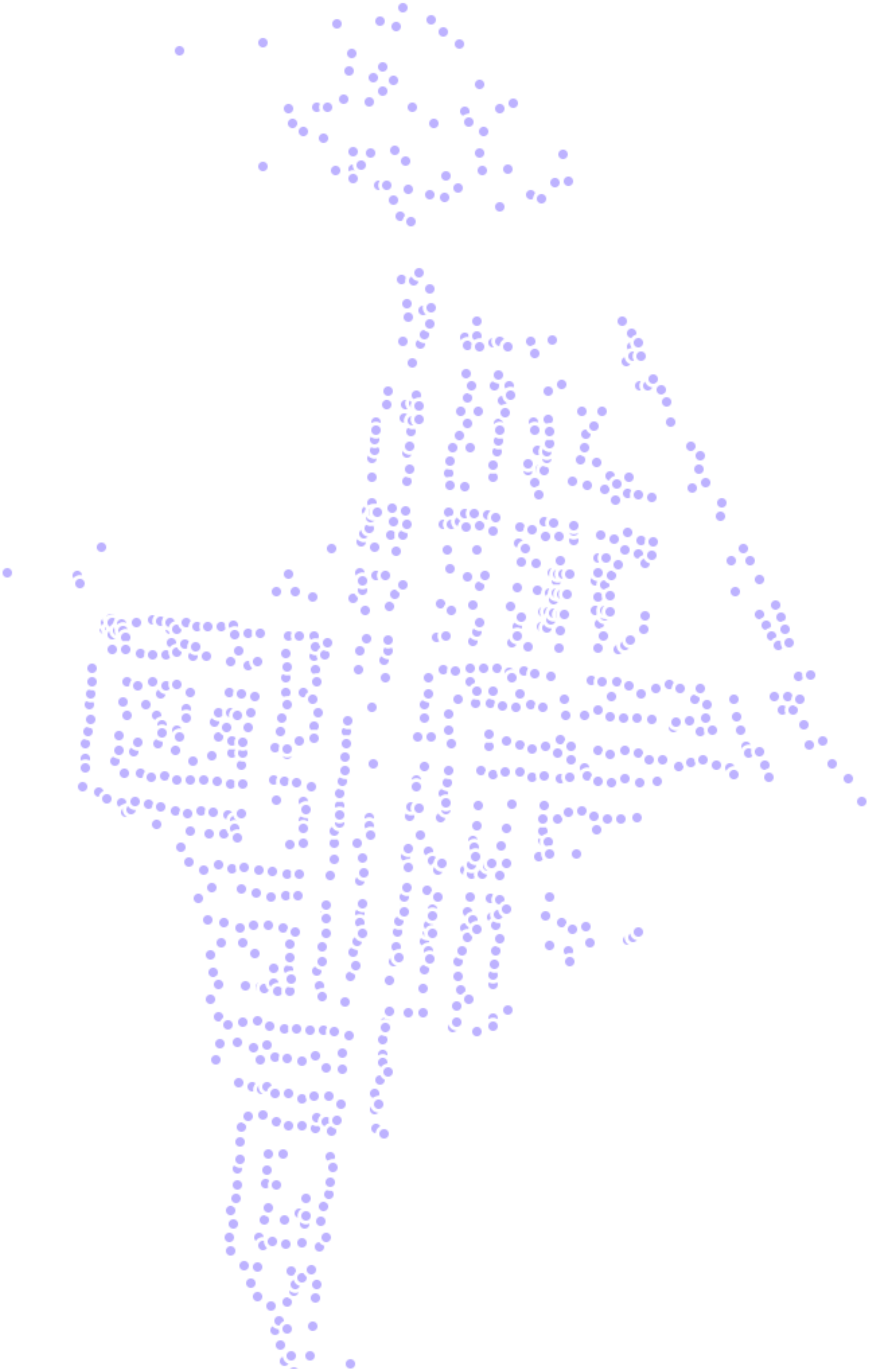

Optimization

This time we will setup two different optimization alternatives: a continuous one (where traps can be placed anywhere in the landscape), and a discrete one (in which traps can only placed in existing sites).

Discrete

The setup for our discrete optimization is pretty straightforward but we recommend the user to have a look at our São Tomé demonstration for more information on the meaning and selection of our parameters.

```
(GENS, GA_SUMSTAT, VERBOSE) = (1000, np.mean, False)
# Parameters for the genetic algorithm internal operation
---------------------
POP_SIZE = int(10*(lnd.trapsNumber*1.25))
(MAT, MUT, SEL) = (
    {'cxpb': .3, 'indpb': 0.5},
    {'mutpb': .4, 'indpb': 0.5},
    {'tSize': 4}
)
```

And let's get our optimization fired up! Please be patient as this part of the code might take around 1h for 500 generations and 16 traps! Once it has finished, go to the **Analysis** part of this notebook.

```
(lnd, logbook) = srv.optimizeDiscreteTrapsGA(
    lnd, verbose=VERBOSE, generations=GENS, pop_size=POP_SIZE,
    mating_params=MAT, mutation_params=MUT, selection_params=SEL,
    fitFuns={'inner': np.sum, 'outer': GA_SUMSTAT}
)
srv.exportLog(logbook, OUT_PTH, '{}D-{:02d}-{:02d}_LOG'.format(ID,
TRPS_NUM, RID))
srv.dumpLandscape(lnd, OUT_PTH, '{}D-{:02d}-{:02d}_TRP'.format(ID,
TRPS_NUM, RID), fExt='pkl')
```

Continuous

In the continuous case we have some variants on the parameters. The mutation is performed by sampling a random number from a normal distribution centered around the allele's current stored coordinate, with the standard deviation needing some tweaking to scale it to the size of our landscape. In terms of the crossover, we select the crossover probability and the "blending" parameter which, when taking a value of 0.5, will do an average between the two parents and assign it to the offspring.

```
(GENS, GA_SUMSTAT, VERBOSE) = (250, np.mean, False)
# Parameters for the genetic algorithm internal operation
---------------------
```

```
POP_SIZE = int(10*(lnd.trapsNumber*1.25))
(MAT, MUT, SEL) = (
    {'cxpb': 0.5, 'alpha': 0.5},
    {'mean': 0, 'sd': 0.0025, 'mutpb': .4, 'ipb': .5},
    {'tSize': 3}
)
# Bounding box for traps position sampling
------------------------------------
delta=0.000015
dPad = (
    (bbox[0][0]*(1+delta), bbox[0][1]*(1-delta)),
    (bbox[1][0]*(1-delta*20), bbox[1][1]*(1+delta*20))
)
```

And that's it. Let's run our GA! Please be patient as this part of the code might take around 1h for 500 generations and 16 traps! Once it has finished, go to the **Analysis** part of this notebook.

```
(lnd, logbook) = srv.optimizeTrapsGA(
    lnd, verbose=VERBOSE,
    bbox=dPad, pop_size=POP_SIZE, generations=GENS,
    mating_params=MAT, mutation_params=MUT, selection_params=SEL,
    fitFuns={'inner': np.sum, 'outer': GA_SUMSTAT}
)
srv.exportLog(logbook, OUT_PTH, '{}D-{:02d}-{:02d}_LOG'.format(ID,
TRPS_NUM, RID))
srv.dumpLandscape(lnd, OUT_PTH, '{}D-{:02d}-{:02d}_TRP'.format(ID,
TRPS_NUM, RID), fExt='pkl')
```

Analysis

We're now ready to take a look at the analysis part of our pipeline. The results will vary slightly depending on if we ran the **discrete** or **continuous** GA alternative, but either way, lets have a look at the evolution of the system:

```
log = pd.DataFrame(logbook)
log.rename(columns={'median': 'avg'}, inplace=True)
(fig, ax) = plt.subplots(1, 1, figsize=(10, 5), sharey=False)
ax.set_title("GA Optimization")
ax.set_xlabel("Fitness")
ax.set_ylabel("Generations")
srv.plotGAEvolution(
    fig, ax, log,
    colors={'mean': '#ffffff', 'envelope': '#1565c0'},
    alphas={'mean': .75, 'envelope': 0.5}, aspect=1
)
ax.set_aspect(.1*(GENS/max(log['avg'])*2))
ax.set_ylim(0, max(log['avg'])*2)
fig.savefig(
```

```
    path.join(OUT_PTH, '{}D-{:02d}-{:02d}_GA.png'.format(ID, TRPS_NUM, 
RID)),
    facecolor='w', bbox_inches='tight', pad_inches=0.1, dpi=300
)
```

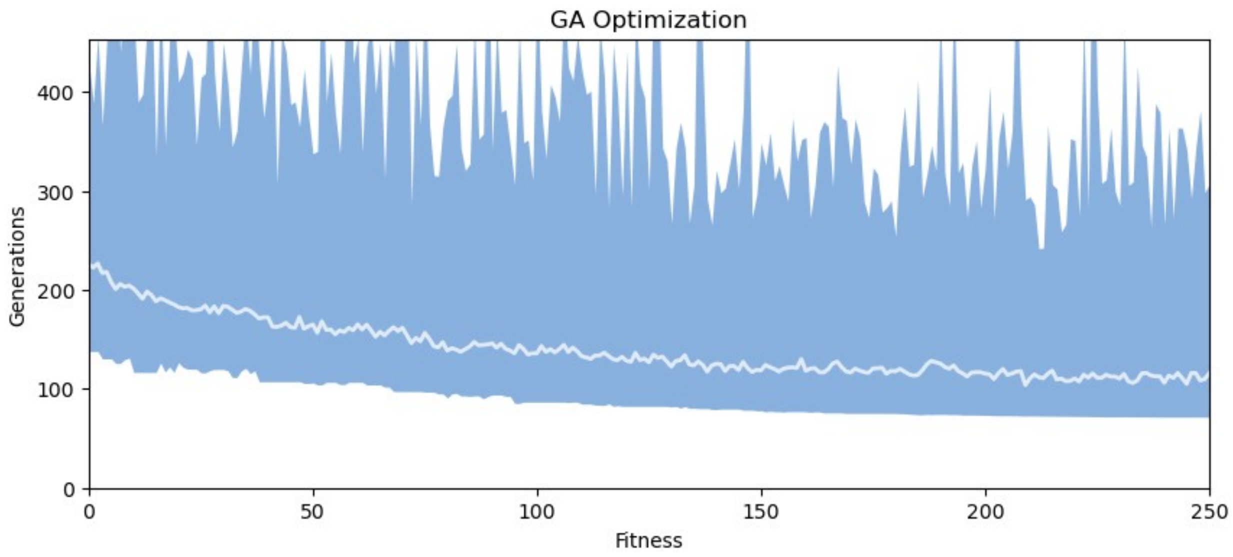

And at our optimized landscape!

```
lnd = srv.loadLandscape(
    OUT_PTH, '{}D-{:02d}-{:02d}_TRP'.format(ID, TRPS_NUM, RID),
    fExt='pkl'
)
(fig, ax) = (
    plt.figure(figsize=(15, 15)),
    plt.axes(projection=ccrs.PlateCarree())
)
lnd.updateTrapsRadii([0.250, 0.125, 0.100])
lnd.plotSites(fig, ax, size=50)
lnd.plotTraps(
    fig, ax,
    zorders=(30, 25), transparencyHex='55',
    proj=ccrs.PlateCarree()
)
srv.plotClean(fig, ax, bbox=lnd.landLimits)
fig.savefig(
    path.join(OUT_PTH, '{}D-{:02d}-{:02d}_TRP.png'.format(ID, 
TRPS_NUM, RID)),
    facecolor='w', bbox_inches='tight', pad_inches=0.1, dpi=400
)
```

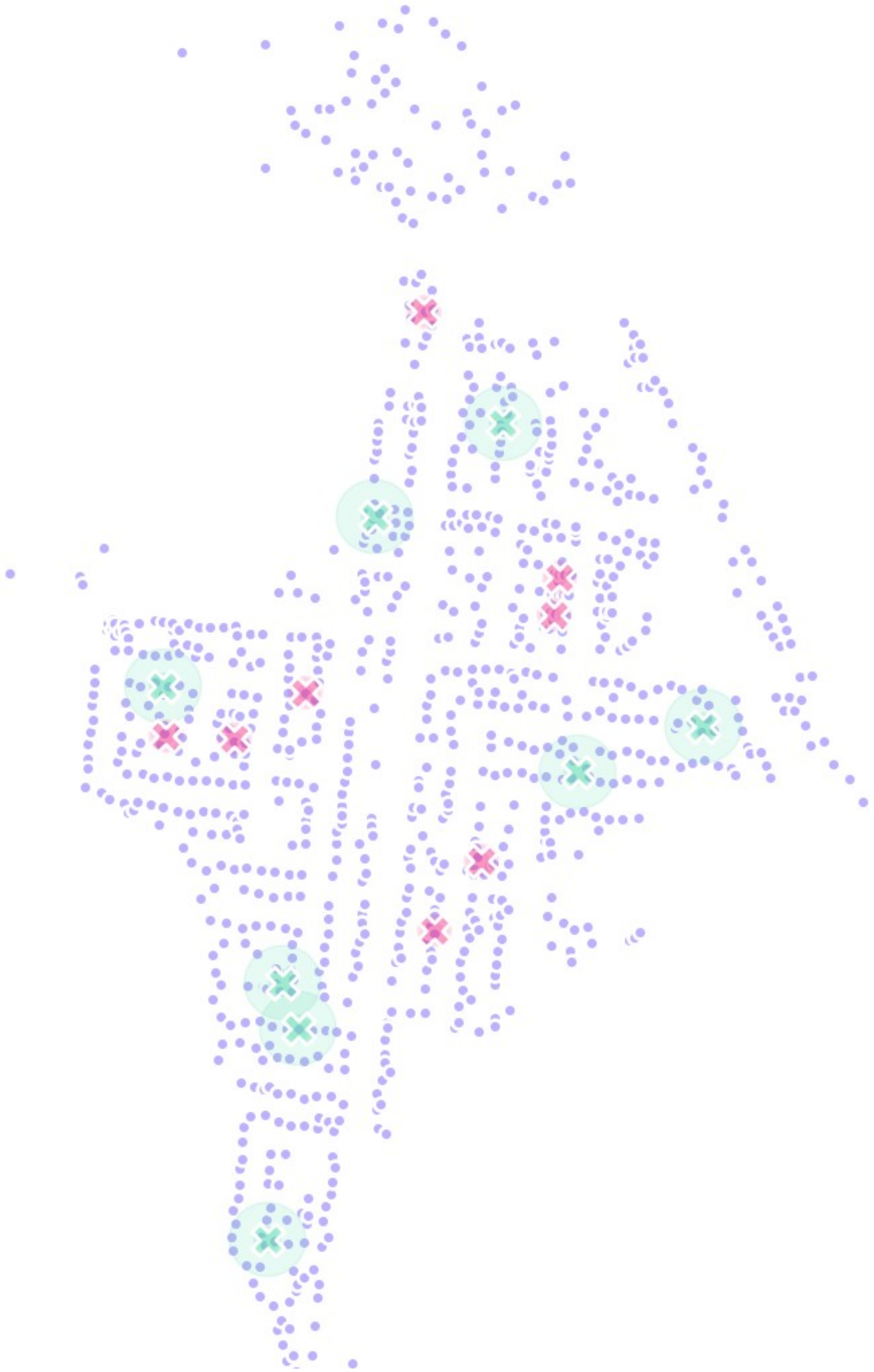

Supplement: S2 Text — Code used to optimize trap placement for Ae. aegypti in Queensland, Australia and An. coluzzii in São Tomé, São Tomé and Príncipe, as described in the manuscript. (PDF) [file pcbi.1012046.s002.pdf]
