# Supplementary material for: MGSurvE: A framework to optimize trap placement for genetic surveillance of mosquito populations
Source: PLoS Comput Biol. 2024 May 6;20(5):e1012046. doi: 10.1371/journal.pcbi.1012046 (PMC11098508; doi:10.1371/journal.pcbi.1012046)
Supplement: Attachment — Submitted filename: MGSurvE-ResponseLetter.pdf [file pcbi.1012046.s003.pdf]

UNIVERSITY OF CALIFORNIA, BERKELEY
Divisions of Biostatistics & Epidemiology
School of Public Health
2121 Berkeley Way #5302
Berkeley, CA 94720

December 18, 2023
To: Editorial Board, PLoS Computational Biology

We appreciate your and the reviewers' time in providing valuable comments that have helped us improve our manuscript substantially. The authors have gone through all the comments and we have done our best to address them all. We hope the manuscript meets the quality standards of your journal, and we welcome any further comments as required. Below we provide the point-by-point responses. All modifications in the manuscript have been highlighted in green (additions) and red stike-out (deletions).

We would like to thank the editorial board in advance for taking the time to consider our submission and look forward to hearing your response.

Sincerely,

Dr Héctor M. Sánchez C., on behalf of the authors
Assistant Project Scientist
University of California, Berkeley
sanchez.hmsc@berkeley.edu

Reviewer 1

This manuscript describes a new software package that the authors have developed to assist researchers in planning field experiments of genetics-based mosquito control strategies. The software runs an algorithm to optimise trap placement for a given scenario, based on inputs including trap type and point locations relevant to the mosquito population of interest (e.g. house locations for the urban dwelling, short dispersing *Aedes aegypti*, village locations for longer dispersing *Anopheles gambiae*). The primary motivation is to support field testing of mosquitoes using gene drive technology to reduce the potential of the target population to transmit disease. This is a somewhat niche application - no gene drive mosquito has yet been field-tested, though several candidates for field testing are in active development - though trap placement and population monitoring more generally is a very important consideration for those working in this area. This is a potentially very useful tool for the audience it is aimed at, though we have a number of comments and suggestions that we feel might improve its clarity and effectiveness. These comments are based on both reading the manuscript and our experience of installing and running the worked examples.

General comments

1. It could be more clearly stated that this tool is to optimise the monitoring of a closed population (e.g. that on an island). A version that is able to optimise trap placement to monitor the spread of a transgene in an open population would be a valuable extension (though perhaps out of scope at this time).
   Thanks for the suggestion. Yes, this is a closed-population optimization tool for the time being but our group is already working in extensions to make it work for larger landscapes by using clustering and aggregation techniques. We have added a small clarification in the last paragraph of the introduction.

2. For one or both of the worked examples, it would have been particularly useful to have a results section with analysis of how much is gained from each additional trap. e.g. number of traps on the x-axis, and time to observe y fraction of mosquitoes on the y axis.. This kind of analysis is something a field-test planner might want to do, so showing how the package can be used this way would help to illustrate its usefulness.
   Thanks for the comment. In the São Tome example we provide the effect of adding traps in increments of five on the average time to capture (shown in the number on the lower right of the figure). We have added text on the figure to clarify the meaning of this quantity. To be able to compute the number or fraction of mosquitoes caught, a model with life-history (such as MGDrivE) would be required, as MGSurvE is aimed at positioning the traps to minimize the time to capture based on Markovian movement. Our research group is currently working on a publication involving the use of MGSurvE's positioning of traps in tandem with MGDrivE in efforts to predict the time to detection of transgenes (as described in the final paragraph of the introduction).

Medium comments

Clarity of mosquito model description

1. We have assumed from the text that the mosquito model acts on a discrete timescale with daily increments? Is this right? Please make this explicit (apologies if we missed it!).
   The software does run in discrete timescales and the examples provided were calibrated to a daily times-step but could be used in different discrete time increments if the migration matrices were calibrated accordingly. We refer to this in the "daily per-capita" movement definition of the movement probabilities (in our "Landscape Specification" paragraph) but added another clarification in the same region of the manuscript to make it clearer (line 85). We have also added labels to our figures to make the units stand out.

2. Are we right to understand that the probability that a mosquito disperses from a to b is dependent on the density of other sites in the vicinity of the focal site (because the probabilities are normalised by their sum)? What you are describing are thus *relative* movement probabilities? Please clarify in the main text (lines 79-96).
   It is an accurate interpretation. The probability of a mosquito moving from a focal point to any other location depends on the availability of alternative places to move towards and their distance from the initial location so, it is a relative movement probability. We have added a clarification in line 86 of the manuscript.

3. Are we right to understand there is no assumption on population sizes – they are implicitly assumed to be equal? If some nodes have larger populations than others, will this affect the results? (I suppose not if it is assumed that the probability of dispersing is not dependent on population size?).
   Yes, it is one of the current simplifications of our software: all of the nodes are assumed to have the same population; so there's no crowding-effect driven migration or optimization focus on populations with larger sizes. We have included in the software, however, a "priority" vector so that the optimization algorithm weighs some specific nodes as more important but it is still going to be tested for versions coming down the line (if one of the nodes, for example, had a significantly larger amount of mosquitoes as compared to the others). We have added a note on this in the "Landscape Specification" section of our manuscript.

Clarity of algorithm description

The genetic algorithm used to optimise trap placement needs to be explained in a bit more detail and clarity for the benefit of those of us not versed in genetic algorithm theory. In non-specialist language, what does the algorithm actually do?

1. A number of words from GA theory are used without clear explanation of what they mean in this specific context. In particular: "Chromosomes", "mutation", "selection", "crossover". Please elaborate on these, taking care to avoid confusion given the biological nature of the application!
   Thanks for your comment. We have re-written the GA section of the paper to make the application and terminology clearer. In doing so, we have also added another section in

the S1 text with thorough information on how MGSurvE uses the GA paradigm in finding solutions to the optimization problem.

2. For a given arrangement of traps, how is the fitness function calculated? Is it deterministic or estimated from stochastic simulations?
   The fitness function is calculated deterministically for each traps' arrangement (although the GA is stochastic itself). It is calculated by solving the Markov chain's fundamental matrix (solving equations 7 and 8 from S1). We have improved the writing in the mathematical section of the S1, and added a thorough description of the GA in the same document to do a better job in describing the details of how our software calculates this metric.

3. How is convergence of the algorithm evaluated/monitored? What is a typical number of iterations needed? Does it give the same (or very similar) outputs on each run?
   The convergence of the algorithm for GA's is usually evaluated by plotting the best achieved fitness in the population of solutions against the number of generations for which the algorithm has been run (lower panel of Figures 3 and 4). The number of iterations, and the repetitiveness of the convergence depend heavily on the evaluated landscape (smaller and more regular landscapes having a higher level of precision). This is now described in the new section of the S1.

4. How efficient is it - how many sites could it handle before becoming unwieldy?
   This is a bit of a difficult question to answer as it largely depends on the available computational resources and the expected run-time. This being said, however, as the matrices scale as the sum of the number of sites and traps, each point makes the optimization process more resource intensive. In our tests we have been able to run landscapes as large as 3800 sites with 50 traps in the timespan of a week of computing time in a server with 44 physical cores (two "Intel(R) Xeon(R) CPU E5-2696 v4 @ 2.20GHz") and 256Gb RAM. Examples such as the São Tomé (around 230 nodes with 20 traps) are manageable under most personal computers in the order of a day or two (computing different stochastic iterations of the optimization cycle). To answer the question a bit more concretely, we have added a factorial benchmark on the speed of a combination of optimization tasks in increasing number of traps and sites. The resulting heatmaps are available now in our software's documentation (https://chipdelmal.github.io/MGSurvE/build/html/benchmarks.html).

Package installation/testing

1. We installed the package on a windows 10 x64-based PC. This was successful when following the "Uneventful instructions", after installing cartopy and libpysal additionally – we felt the documentation could be a bit clearer with respect to installing these additional packages.
   Glad to hear that the installation went well, albeit with a couple of minor hiccups. We added a bit more description on MGSurvE's dependencies and how they interact with our software in our website so that it is easier for everyone to get the software up and running (https://chipdelmal.github.io/MGSurvE/build/html/installation.html).

2. We tried running the examples from the paper. At first we had a little trouble before learning that we needed to download the csv data point files – the README in the github demo folder could be clearer about the need to download the csv files if not running from bash.

Thank you for the comment and suggestion. We have added instructions to run the paper's examples in their respective README files: https://github.com/Chipdelmal/MGSurvE/tree/main/MGSurvE/demos/STP and https://github.com/Chipdelmal/MGSurvE/tree/main/MGSurvE/demos/YKN.

3. YKN discrete takes very long to run, so we did not run it all! We had more success with the "Quickstart tutorial", which took a few minutes.
   The Yorkey's Knob example does require a sizeable amount of computational resources. It can be run on personal computers but they need a large amount of RAM memory to allocate the migration matrix, along with at least a couple of CPU cores to run the computations in parallel (better suited for a dedicated server). We have added a new section in our documentation to give the users a better sense of the computational resources needed to run full-fledged models in their computers (https://chipdelmal.github.io/MGSurvE/build/html/benchmarks.html).

Minor comments

1. Fig. 1. Where it is first mentioned in the text, it could be clearer that the figure describes the structure of the method and not the visualisation itself.
   Thanks for the note. We have moved the reference to Fig. 1 earlier in the paragraph to make it clearer that it refers to the three components, rather than the visualization.
2. Fig. 3. The figure legend describes the plots in a different order to the figure itself! (B,C,A).
   The figure legend has been re-ordered to describe plots in the same order as they are shown (A, B, C).
3. Fig. 3. C needs more explanation - the two axes need some elaboration. NB 'generations' and 'fitness' obviously have resonance to both both the mosquito biology and GA terminology, so there is potential for confusion. Also the x-axis seems way over-stretched, only really interesting in the lower range.
   We appreciate the comment. We have changed the labels for the axes to be more specific, added more explanation in the caption, and reduced the range on the x-axis panels for figure 3.
4. Sao Tome example. A daily (? or generational?) staying probability of 0.05 of staying in a village seems very low!!?
   This is a mistake on the description of the experiment (Fig 4). In the earlier stages of development we were using a daily stay value of 0.05 for debugging purposes but we later updated to the realistic parameter of daily staying higher than 0.97 (values along the diagonal of the migration matrix used in the experiments https://github.com/Chipdelmal/MGSurvE/blob/main/MGSurvE/demos/STP/GEO/STP_MigrationN.csv). We apologize for the error and have corrected the text to reflect the experiments.
5. Line 227. I don't think 'amplitude' is a conventional word for describing this kind of dispersal kernel, and unclear what it means..?
   Thanks for the comment. We have changed the term "amplitude" for "maximum attractiveness" to match the terminology used in the figures and throughout the text. We have added an explanation of the term in Figure 2's caption (where the term first appears), and there's some further clarification in line 186 where we describe the traps used for the suburban landscape application.

6. Fig. 2 could benefit from a colour scale bar? And a legend for the map symbols. On the matrix (C), spell out r and g (red and green, presumably).
   Thanks for the suggestions. We have added a color bar to the matrix plot, legend for the map symbols, and “r/g” labels in the traps on the maps to match the labels on the matrix plots.

7. Figs 3 and 4. Put the plot labels A and C in the top left corner, hard to notice them where they are!
   Labels in figures 3 and 4 have been moved to the top-left corner (outside).

8. What are the little numbers at the bottom right of each map plot?
   The numbers at the bottom right of the plots represent the best achieved fitness in the optimization (the expected mean or maximum time it takes for one of the random walkers to fall into a trap provided they started anywhere on the landscape). We have added a corresponding label in the figures.

9. We didn’t find the supporting videos very instructive at all. They are very slow and with no narration it is hard to see what is going on. Perhaps they could show how the inputs are used, and include some narration of what is going on?
   Thanks for pointing it out. We agree with this remark. We have removed the original videos from the publication and replaced them with a reference to the online webinar we held some weeks after we received the manuscript’s feedback (both figures now point to the S1 Webinar video with their timestamps).

Reviewer 2

The manuscript presents MGSurvE, a framework to investigate optimal trap placement. Optimal trap placement is an essential task for many applications of genetic monitoring. The manuscript was mostly easy to read for people with a background in genetic algorithms and mosquito dispersal. But sometimes the manuscript felt repetitive to read and incomplete. Some sentences are too complex and challenging to read, e.g. lines 3-6 or Fig 2 lines 2-5. Split them up into multiple sentences for an easier read.

Comments

1. Line 11-12; what is split dive? Can you explain in a sentence how it works. How is it different from gene-drive.
   Thanks for the remark. A clarifying sentence has been added to the manuscript to explain the split drive system (line 12).

2. Line 71-72; how much is local dispersal and greater distance? Can you provide an approximate distance from the reference [19] and [20] in the text
   Thanks for the clarification request. We have added a note on the suggested lines stating the general expected movement range for Aedes *(on the order of meters), and* Anopheles *(usually on the order of around five kilometers).*

3. Line 73-77; You claim that MGSurvE can represent specific resources e.g. blood, sugar, and water, but none of your examples in the manuscript uses the biological modifying parameters. There is also no definition in the “S1 Text: Model description” of how the “biological modifying parameters” or “sex-specific” modify the movement.
   Thanks for the comment. Specifying this information would be important in situations in which we think resource heterogeneity would play a major role in mosquito movement in the landscape under study (where there is resource scarcity, for example). We didn’t include this information in the manuscript examples as we do not have enough information to parameterise a study that went into the finer details of mosquito movement. We do, however have examples in our documentation where we setup and run synthetic cases taking spatial heterogeneity (https://chipdelmal.github.io/MGSurvE/build/html/GA_sites.html) and sex-specific kernels into account (https://chipdelmal.github.io/MGSurvE/build/html/GA_complex.html). We have added a line refering to this in the document (in Yorkey’s Knob results section), and added the description of the biologically-modifying parameters in the S1 document.

4. Line 88; a figure in the supplement showing the different types of kernels of mosquito movement or trap attractiveness would be helpful for readers with a biological background.
   Thanks for the suggestion. We have added a plot on the mosquito movement kernel in S2, along with the function that produces it. We have also added plots on the traps attractiveness and their respective code for both examples in S2.

5. Line 111-144; The provided explanation of the genetic algorithm (GA) is only understandable for people who know how GA works and even then it is hard to understand. How are the traps on the chromosome as allele optimized? Can you provide a figure in the supplement for easier understanding?

Thanks for the comment. We have re-written this section to make it more appealing to the readers, and we extended the S1 with a thorough section on how the Genetic Algorithm is encoded in the MGSurvE framework. Several diagrams and explanations on each part of the process (fitness, mutation, crossover, selection, etc) are now provided.

6. Line 171; the reference [28] for local disperser differs from the reference [19] mentioned on line 71. Why do you have two different references at two different locations in the manuscript? Should the reference be cited together if they both report the local dispersal of A. aegypti? Again can you provide an approximate distance from the reference?
   Thanks for noticing. Both references are now in the first mention of the flight distance of the A. aegypti *mosquito along with a note on the order of magnitude of the expected movement range.*

7. Line 191-202; I missed a summary of the optimization results in the text. No information about the optimization parameter in the main text is provided e.g. how many generations and replicates were used to get the results. How many traps and why that many traps? What was the mean and max time expected time for a given mosquito to be trapped? Why are the references to the videos not mentioned in the mean text? It feels odd that the Fig 3 text provides more results information than the main text.
   Thanks for the comment. We have added a new paragraph to the experiment description with analysis of the results, and moved some of the information from the caption to it.

8. Figure 3; It would be nice to have a figure legend in the plots. Figure labels A, B1-3, and C are hidden in the figure and should be located outside of the figure in black. What is the meaning of the colors in Figure 3C? What is the meaning of the grey number in Figure 3B? What was the optimal number of traps? Move the reference to the videos to the main text as it does not describe anything in the figure.
   We have added legends to panels A and B to make them clearer, as well as callouts from panel B to C to connect the maps to their optimization processes. We also moved the figure labels to outside the frame in black. The number in the maps has been labeled as the fitness, and the reference to the videos has been updated.

9. Line 212; Why does the resource type not needed to be specified, explain more clearly what the scale of the localities means, e.g. due to the 500m minimum distance between nodes all the resources are provided within each node.
   Thanks for the question. In Sao Tome's case, the aggregation of the households make it so that mosquitoes should have all the resources needed for their lifecycle (otherwise, there would be no mosquitoes in the area, and no need to take it into account in the surveillance scheme). We have added a clarification note in the demonstration's text.

10. Line 205-207; Why is more information about the study side e.g. 'west of the cost Gabon' provided in the Figure 4 text than in the main text?
    Thanks for the comment. We have modified the caption to cut the information that was redundant to the one in the main text, and we have moved the additional one to outside the figure caption.

11. Line 228-240; I missed a summary of the optimization results in the text. What was the optimal number of traps? Also same issues as in point 7: No information about the optimization parameter in the main text are provided e.g. how many generations and replicates were used to get the results. What was the mean and max time expected time for a given mosquito to be trapped for the different number of traps. Why are the references to the videos not mentioned in the mean text?

Thanks for the comment. We have added a new paragraph to the experiment description with analysis of the results, and moved some of the information from the caption to it.

12. Figure 4; The two immovable trap is not described in the figure text. It would be nice to have a figure legend in the plots. Figure label A, B1-3, and C are hidden in the figure and should be located outside of the figure in black. What is the meaning of the colors in Figure 4C? What is the meaning of the grey number in Figure 4B? Move the reference to the videos to the main text.
Thanks for your recommendations. We have added a legend for the immovable and movable traps with a reference in the figure's caption. Additionally, we added callouts to the colors of the plots so that it is clear that they match the number of traps in the landscape, and the fitness label to the number underneath the map.

13. What type of application do you need to specify different types of resources? Why is there no example of different types of resources? One of the examples could have been modified to use the different resource types. It would have been interesting to see the difference in the optimal trap placement.
Thanks for the suggestion. Specifying this information would be important in situations in which we think resource heterogeneity would play a major role in mosquito movement in the landscape under study. We didn't include this information in the manuscript examples as we do not have enough information to parameterise a study that went into the finer details of mosquito movement. We do, however have examples in our documentation (`https://chipdelmal.github.io/MGSurvE/build/html/GA_sites.html`), where we setup and run synthetic cases taking spatial heterogeneity and sex-specific kernels into account (`https://chipdelmal.github.io/MGSurvE/build/html/GA_complex.html`). We have added a line referring to this in the document (in Yorkey's Knob results section).

14. I would have liked to see an overview of different used kernel definitions for traps (mentioned in line 100) and mosquito dispersal in the supplement.
Thanks for the comment. We didn't generate a thorough sensitivity analysis demonstration for the current examples as our goal was to show some use examples of our software package. We do, however, use different movement patterns in the two different examples and we use two different types of traps in our Queensland demo. Additionally, S2 now shows readers how everything is setup and makes it easy for them to modify parameters as needed through the downloadable jupyter notebooks in the github repository.

15. Line 261-262; Could you elaborate in the text on what would change when taking mosquito life history and intergenerational movement into account? Would only the time change or also the optimal trap placement? Is it really necessary to include the mosquito life history for optimal trap placement?
Thanks for the note. We agree that, for most applications, the result of adding life-history would probably return the same results albeit with changes in the time-to-trap change. There could be situations, however, in which, given seasonality, changes in the distribution of resources, or crowding effects, mosquitoes could migrate in different ways from the ones we would initially assume if we only used a random-walker simplification for the optimization.

16. Line 242; Include the DOI (10.5281/zenodo.8087603) of the software version used in the manuscript
Thanks. The DOI url was added to the manuscript at the suggested line (`https://doi.org/10.5281/zenodo.8084253`).

17. What is the unit of the expected number of time steps, e.g. days, weeks, generations?
    In the examples provided in the document, the time-steps are measured in days (as that's how the movement kernels were calibrated). We have added labels to the figures to make this more apparent.

Typos

1. 'S' missing In author summary without line numbering approx on line 4: are considered aS novel tool
   Thanks for the comment. In this sentence we are referring to the concept of gene drives as a whole, and stating that the concept is considered a novel tool in the field, so we believe the sentence is correctly phrased as is.

S1 Text: Model description

1. The online documentation is much more appealing than the model description. The model description would benefit if the information in the "Pkg Breakdown" section were included alongside the formulas in the model description.
   We added a new section in the S1 in which we go into more detail about how the GA is implemented in the package, along with references to the equations in the mathematical description of the document and new diagrams to explain the whole optimization process.
2. How does the biological modifying parameter modify the shape parameter?
   These parameters are part of the traps attractiveness kernel and control how the main function takes shape. For example, if the attractiveness is exponentially-shaped, the ρ would be the decay rate. We have added a description on line 12 of the S1 document to clarify.
3. Multiple parameters are not defined in the text or do not appear in the formulas, e.g. tj, tj, $trap$
   Thanks for the remark. We have added the s_i and t_j on the description, and changed the $trap$ in equation 3 to match the nomenclature. We also added an extra parragraph after equation 6 explaining each element of the block matrix.
4. Typo: remove 'g' in "Markov chainG properties"
   Typo was removed (line 29 of S1).

S2 Text: Example use case

1. For x0 and b use the distance in meters for easier understanding, e.g. 1/16 = 0.0629, or define from where the value 0.0629534 originates.
   Thanks for the note. The specification of the parameters is now stated in meters (16 m) with the exponential and sigmoid kernels definitions being derived from this value.
2. I miss the definition of global parameters, e.g.`TRPS_NUM` and `GENS`
   We have converted S2 into a set of interactive jupyter notebooks for the São Tomé (https://github.com/Chipdelmal/MGSurvE/blob/main/MGSurvE/demos/STP/STP-Discrete.ipynb)

and Yorkeys Knob (`https://github.com/Chipdelmal/MGSurvE/blob/main/MGSurvE/demos/YKN/YKN.ipynb`) demonstrations. These were updated with a more thorough description of the used constants and parameters. A PDF version of these notebooks is now the S2 document.

3. What is the difference between `optimizeTrapsGA` and `optimizeDiscreteTrapsGA`. I assume continuous and discrete optimization. Clarify that in the "Yorkey's Knob" example. *Yes, we coded both versions of the optimization task for both examples even if we only showed the continuous one for Yorkey's Knob. The continuous version is now available on the S2 document as well as in the jupyter notebook demonstration code.*

4. Many parameters are not described, e.g. `pop_size='auto'`, *These parameters are now provided as an explicit dictionary, and are better described in the Jupyter notebooks version of our demos.*

5. Examples contain errors in the trap type specification of Sao Tome in tKer params 'b'. *Thanks for pointing the discrepancy out. We have corrected this error, and updated it with the right value and followed the previous advice of using the mean distance as the parameter value.*

6. What does the function deepcopy? *This function is part of base python and, in general, creates a copy of the object being passed as an argument including copies of all the underlying objects referenced by the entity (lists, sets, variables); so that the copy doesn't point towards the same entities (in memory) the original object did (more information can be found in: `https://docs.python.org/3/library/copy.html`).* We have removed its use from the examples, as it was not strictly needed.

Reviewer 3

This manuscript introduces and describes MGSurvE, a software developed to optimize placement of ovitraps to detect mosquito populations with the goal of early detection of alleles, either those that are introduced or those that may be resistant or antagonistic to introduced genes. The manuscript is a well-written description of the software, and the authors present results for two scenarios in particular for which the software may be useful. The software is clearly important to trap placement and design of experiments that involve trap placement. I only have a few comments and questions about the manuscript in its current form.

1. The authors mention integration of MGSurvE with other software developed by members of this group. In particular, the authors write "MGSurvE may be used in parallel with MGDrivE or MGDrivE2…" Could the authors elaborate on this with a few sentences? Either here or later in the manuscript? In particular, how do the authors see this software working parallel with the others? Is there integration between them? Additionally, do the authors think that MGSurvE may be integrated with other complex models of gene drive systems other than the MGDrivE models?
   MGSurvE can export migration matrices that are easily usable in our MGDrivE frameworks. This is achieved through the $exportLandscape$ function (`https://chipdelmal.github.io/MGSurvE/build/html/MGSurvE.html#MGSurvE.auxiliary.exportLandscape`), which exports the migration matrix including the traps, along with the site/trap positions and their corresponding types into CSV files. Our MGDrivE frameworks model mosquito movement in a way that is consistent with this information. Specifically, MGDrivE v1 uses daily time-steps by default, so it can readily take the matrix and simulate gene drive spreads in spatially-explicit landscapes; whereas MGDrivE v2 requires some translation of the discrete timestep into movement rates. We have added a line on this in the section the reviewer kindly requested.

2. Given the importance of the optimization of trap distribution to this work, could the authors elaborate more on the optimization process? I think some of the details of process are lost in the use of GA-associated terms, but I think it could be helpful to discuss this section in a way that is more generally accessible in order to underscore the utility of the software to a wider audience. If the goal is to minimize the distance between breeding/feeding sites and traps, what is the cost associated with the optimization? What is the cutoff to decide on the "optimal" solution? As is mentioned later in the manuscript, local optima can be found instead of global optima. How is this dealt with? Additionally, are there differences in the way the GA optimizes over the discrete vs continuous optimization processes? It may benefit the overall clarity of the main text to incorporate some of the mathematical details from S1.
   Thanks for your comment. We have re-written the GA section of our manuscript taking this into account, and we have added a new section on the description of the optimization process and the genetic algorithm to the S1 document which goes into much more detail on how all the operations interconnect in our framework. This new section also addresses the local optima concerns, and the specifics of how parameters were setup for the optimization tasks.

3. In the first paragraph in the Queensland example, the authors mention that "Resource types do not need to be specified, as a household provides all the feeding and breeding resources…" Are there ways in which heterogeneity in feeding and breeding resources

can be considered? For example, one could imagine a home with many people that may provide ample feeding resources for mosquitoes but no breeding resources (or, alternatively, an abandoned home with breeding resources but no feeding resources).
Thank you for the comment. MGSurvE can take into account these resources in situations as the one described by the reviewer, where scarcity and heterogeneity might be important factors to consider. If such information is available, an additional matrix can be fed to the algorithm (this matrix would contain the probability of a mosquito searching for another resource from any other of the available ones). We have added notes on this in the description of the experiments and links to examples in our documentation for the reader to get more information on how to implement these kinds of studies.

4. Availability and Future Directions: Can the authors elaborate on the utility of this sort of model for mosquito trap placement in a broader sense? That is, would this optimization process be useful to someone who is just looking to capture the best possible representation of a local mosquito population through trapping? I can envision interest in this software from entomologists and field ecologists who are studying mosquito population dynamics, particularly those who may have limited resources for trap placement and personnel to monitor traps. Could this model be adapted to include also the "financial costs" associated with placing, managing, and monitoring traps?
   Thanks for the comment. We are currently working on extensions that would take into account financial costs in laying down and monitoring traps. Specifically, we have started working in co-optimizing the positioning of the traps in tandem with the routes that would need to be followed by driving vehicles in order to monitor them, along with the extension that would allow the algorithm to pick the most cost-efficient traps available to the surveillance team. We have added a small paragraph in the "Future Directions" section, as well as our project's website (`https://chipdelmal.github.io/MGSurvE/build/html/FutureDirections.html`).

5. Figure 2C. The representation of this matrix with the Greek letters was initially unclear to me, and I wonder if some additional text in the "Landscape Specification" section could help with interpretation. Presumably, for a population with N locations and M traps, There is an $N*N$ matrix that denotes movement between traps (τ), an $N*M$ matrix that denotes movement from locations to traps (ν), a zero matrix that indicates that mosquitoes located in traps do not go anywhere (0), and an identity matrix that indicates that mosquitoes in traps stay in traps (I). Then, the matrix represented in figure 2C is just all of these combined in one matrix to indicate movement between $N+M$ locations and traps. Is this correct?
   Thanks for the comment. Yes, the matrix shown in figure 2C is a block-matrix in canonical form as described by the reviewer. We have added a legend on the figure with the terms of the matrix and improved our description in the caption with the suggestions so that it's clearer for readers.

6. Figure 3. A very minor comment, but could the scale be changed on the horizontal axis of Figure 3A to better show the differences between the curves? That is, is it necessary for this axis to go beyond 60m?
   Thanks for the remark. We have changed the plot range to span up to 50m to highlight the more interesting region of the visualization.

7. Figure 3C and Figure 4C. Could you clarify what is driving the difference in initial values of these curves? For example, in Figure 3C it seems that there are numerous curves for the continuous mean. How are these different curves generated?

Each one of the “optimization traces” was generated by running a completely independent optimization process on the landscape. Each scenario was optimized X times to test for convergence, to avoid “local optima”, and to check if the GA algorithm was being run for a long enough time. We have added a description of the traces both in the experiments text and figures captions.
